# ELPIS-JP: a dataset of local-scale daily climate change scenarios for Japan

**DOI:** 10.1098/rsta.2011.0305

**Published:** 2012-03-13

**Authors:** Toshichika Iizumi, Mikhail A. Semenov, Motoki Nishimori, Yasushi Ishigooka, Tsuneo Kuwagata

**Affiliations:** 1 Agro-Meteorology Division, National Institute for Agro-Environmental Sciences, 3-1-3 Kannondai, Tsukuba, Ibaraki 305-8604, Japan; 2 Centre for Mathematical and Computational Biology, Rothamsted Research, Harpenden AL5 2JQ, UK

**Keywords:** ELPIS-JP, stochastic weather generator, LARS-WG, climate change, impact assessment, Japan

## Abstract

We developed a dataset of local-scale daily climate change scenarios for Japan (called ELPIS-JP) using the stochastic weather generators (WGs) LARS-WG and, in part, WXGEN. The ELPIS-JP dataset is based on the observed (or estimated) daily weather data for seven climatic variables (daily mean, maximum and minimum temperatures; precipitation; solar radiation; relative humidity; and wind speed) at 938 sites in Japan and climate projections from the multi-model ensemble of global climate models (GCMs) used in the coupled model intercomparison project (CMIP3) and multi-model ensemble of regional climate models form the Japanese downscaling project (called S-5-3). The capability of the WGs to reproduce the statistical features of the observed data for the period 1981–2000 is assessed using several statistical tests and quantile–quantile plots. Overall performance of the WGs was good. The ELPIS-JP dataset consists of two types of daily data: (i) the transient scenarios throughout the twenty-first century using projections from 10 CMIP3 GCMs under three emission scenarios (A1B, A2 and B1) and (ii) the time-slice scenarios for the period 2081–2100 using projections from three S-5-3 regional climate models. The ELPIS-JP dataset is designed to be used in conjunction with process-based impact models (e.g. crop models) for assessment, not only the impacts of mean climate change but also the impacts of changes in climate variability, wet/dry spells and extreme events, as well as the uncertainty of future impacts associated with climate models and emission scenarios. The ELPIS-JP offers an excellent platform for probabilistic assessment of climate change impacts and potential adaptation at a local scale in Japan.

## 1. Introduction

The major factors that have hampered progress in assessment of possible impacts of climate change and adaptation at a local scale are the coarse spatial resolution and systematic errors (called bias) of global climate models (GCMs), and uncertainty of climate projections associated with using different GCMs and greenhouse gas and aerosol emission scenarios. Recently, 20 km grid atmosphere-only GCMs have been made feasible [1]. Yet, the simulation period and the size of ensembles are limited for such GCM experiments, constraining probabilistic impact assessment. To that end, many ensembles of higher resolution and less biased climate data for specific regions (referred to as climate change scenarios) are required for impact studies (e.g. [2,3]). Dynamical and statistical downscaling methods (SDMs) are expected to bridge available GCM outputs and climate inputs required for impact models.

Regional climate models (RCMs) are a dynamical downscaling method that can provide high-resolution and physically consistent climate data derived from a coarse resolution GCM output. RCMs are powerful tools to examine underlying physical reasons for projected change in regional climate and are expected to achieve better representation of extreme events and effects of topography, land use, coastlines and their interactions on regional climate than GCMs [4]. Nonetheless, RCM daily outputs could have a certain bias (e.g. drizzle) and are not directly used as climate inputs for process-based impact models, such as crop models [5,6]. For this reason, in general, SDMs are further applied to RCM outputs to generate climate change scenarios.

Among various SDMs, weather generators (WGs) are a unique tool that can generate many sequences of daily weather data at a specific site without heavy computational requirement. The daily values of a climatic variable are sampled from the probability distribution of climatic variables estimated from historical data at a given site and have similar statistical properties to historical data [7]. By modifying distributions using the information from a climate model, GCM or RCM, WGs can generate large ensembles of daily climate change scenarios at a given site. Owing to such usefulness and proven adequacy in various climates, the LARS-WG weather generator [8] has been frequently used for climate change scenario generation in various regions [3,7,9]–17], although there are a few studies in Asia under a monsoon climate [18,19].

In Japan, some climate change scenarios are provided using the delta method, which adds a difference (or multiplies a ratio) between the future and current climate projections to observed data [20,21], or a bias-correction method [22,23]. A regression method is used for research purposes rather than for scenario generation [24]. However, these scenarios have a limited number of climate models, emission scenarios and climatic variables. For instance, the scenarios developed by Okada *et al*. [21] are the most synthetic ones, but they do not include relative humidity and wind speed or climate projections under the Special Report on Emissions Scenarios (SRESs; [25]) B1, which represents the lowest CO_2_ concentration pathway. For these reasons, more daily climate change scenarios are required for impact studies in Japan.

Following the ELPIS for Europe [3], we developed a dataset of local-scale daily climate change scenarios for Japan (called ELPIS-JP) using the weather generators LARS-WG and, in part, WXGEN with the multi-model ensemble from the CMIP3 [26] and multi-model ensemble of RCMs provided in the S-5-3 project [27,28]. The objectives of this study were to assess the applicability of the WGs in a monsoon climate, specifically Japan, and describe the scenario generation procedure, features and limitations of climate change scenarios, taking the ELPIS-JP dataset as an example.

Section 2 includes a description of the observed daily weather data and climate model outputs as well as a description of the WGs. Evaluation of the WGs is presented in §3. Section 4 describes the scenario generation procedure and the features and limitations of the generated climate change scenarios. Conclusions are presented in §5.

## 2. Data and methods

### (a) Observed daily weather data

**Figure 1.**
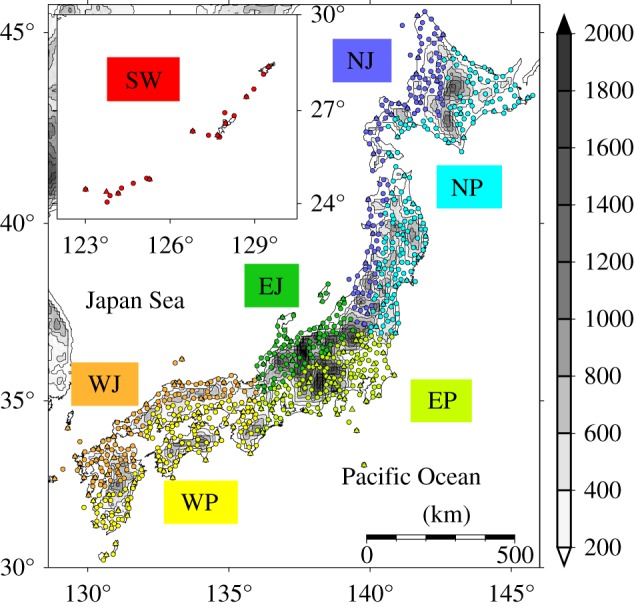
Location of AMeDAS (circle) and JMA (triangle) sites. The areas in Japan are indicated by colour: North Japan/Japan Sea side (NJ), North Japan/Pacific Ocean side (NP), East Japan/Japan Sea side (EJ), East Japan/Pacific Ocean side (EP), West Japan/Japan Sea side (WJ), West Japan/Pacific Ocean side (WP) and southwestern islands (SW). The grey shading indicates altitude above sea level.

The observed daily weather data for the 20 year period 1981–2000 were obtained from the model coupled crop–meteorological database developed at the National Institute for Agro-Environmental Sciences (called MeteoCrop DB; [29–31]): this database includes the estimates of variables, such as solar radiation and water temperature, simulated by various models, as well as the observed data. The MeteoCrop DB includes daily mean, maximum and minimum temperatures (*T*_ave_, 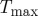 and 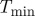), precipitation (Pr), solar radiation (SR), relative humidity (RH) and wind speed (WS) observed at 783 Automated Meteorological Data Acquisition System (AMeDAS) sites and 155 surface observatories maintained by the Japan Meteorological Agency (JMA). For daily mean WS, it was corrected to the value at 2.5 m above ground using the aerodynamic roughness length. These observation networks densely cover the whole of Japan, including surrounding islands (figure 1).

The JMA sites observe all seven climatic variables, whereas the AMeDAS sites do only *T*_ave_, 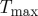, 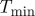, Pr, WS and sunshine duration. In the MeteoCrop DB, the daily total SR value at the AMeDAS sites was estimated from observed sunshine duration by using the modified version of the Ångström–Prescott equation [32–34] that incorporates the adjustment of sunshine recorder coefficients to reduce the instrumental bias in observed sunshine duration. The daily mean RH value at the AMeDAS sites was estimated using the spatially interpolated RH value at the nearest neighbouring JMA sites. In the multi-site average, the percentage of missing data for the 20 year period was less than 0.2 per cent at maxima (Pr), indicating the excellent quality of the database to estimate the WG site parameters.

### (b) Climate model outputs

Table 1 summarizes the climate models used for the ELPIS-JP dataset. All seven climatic variables (*T*_ave_, 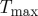, 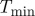, Pr, SR, RH and WS) were available from most GCMs. The GCM transient monthly outputs for the 130 year period 1971–2100 were obtained from the CMIP3 database [26]. Only one representation of climate was used for each GCM. Monthly mean daily maximum and minimum temperatures were estimated by adding (or subtracting) half of the observed climatological diurnal temperature range obtained from the TS 2.1 dataset of the Climate Research Unit, University of East Anglia [35], UK, to the GCM monthly mean temperature, if the GCM outputs for these variables were not available. In the ELPIS-JP dataset, the climate change scenarios derived from the GCMs accounted for the mean climate change, but did not account for changes in climate variability, including wet/dry spells, because no GCM daily outputs are readily available for the whole period. Only for the high-resolution version of the Model for Interdisciplinary Research On Climate v. 3.2 (MIROC-H) under the A1B scenario, were the changes in climate variability and wet/dry spells accounted for based on the GCM daily outputs obtained from the database developed at the Centre for Climate System Research (CCSR), University of Tokyo, Japan.

**Table 1.** General information on the climate models used for the ELPIS-JP dataset.

model acronym	CMIP3 model designation	research centre(s)	country	emission scenarios	source(s)	note
GCM (global, the grid interval varies by model and by 1.1–5.0^°^ in longitude)						
BCCR	BCCR-BCM2.0	Bjerknes Centre for Climate Research	Norway	A1B, B1, A2	Déqué [36]	mean change only
CGCM-T47	CGCM3.1(T47)	Canadian Centre for Climate Modelling and Analysis	Canada	A1B, B1, A2	Flato *et al*. [37]	mean change only
CGCM-T63	CGCM(T63)			A1B, B1		mean change only
FGOALS	FGOALS-g1.0	Institute of Atmospheric Physics	China	A1B, B1	Wang *et al*. [38]	mean change only
GISS-AOM	GISS-AOM	Goddard Institute for Space Studies	USA	A1B, B1	Russell *et al*. [39]	mean change only
INMCM3	INM-CM3.0	Institute for Numerical Mathematics	Russia	A1B, B1, A2	Galin *et al*. [40]	mean change only
IPSL	IPSL-CM4	Institute Pierre Simon Laplace	France	A1B, B1, A2	Hourdin *et al*. [41]	mean change only
MIROC-H	MIROC3.2(hires)	Centre for Climate System Research/National Institute for Environmental Studies/Frontier Research Centre for Global Change	Japan	A1B, B1	K-1 Model Developers [42]	mean change only^a^
MIROC-M	MIROC3.2(medres)			A1B, B1, A2		mean change only
MRI	MRI-CGCM2.3.2	Meteorological Research Institute	Japan	A1B, B1, A2	Yukimoto & Noda [43]	mean change only
RCM (over Japan, the grid interval is 20 km)						
NHRCM	—	Meteorological Research Institute	Japan	A1B^b^	Saito *et al*. [44], Ishizaki & Takayabu [45]	mean and variability changes
NRAMS	—	National Research Institute for Earth Science and Disaster Prevention	Japan	A1B^b^	Pielke *et al*. [46], Dairaku *et al*. [47–49]	mean and variability changes
TWRF	—	Center for Computational Sciences, University of Tsukuba	Japan	A1B^b^	Skamarock *et al*. [50]	mean and variability changes

^a^Mean and variability changes scenarios are available for A1B.

^b^Initial and lateral boundary conditions of the RCM were obtained from the MIROC-H output (20C3M and A1B).

The multi-RCM outputs provided in the S-5-3 project were used for the ELPIS-JP dataset as well as for the GCM outputs. Three RCMs listed in table 1 were non-hydrostatic models and used different physical parametrization packages for cumulus convection, microphysics, planetary boundary layer and land surface process. The RCMs covered the whole of Japan with a common grid interval of 20 km and had nearly common centre pole positions of the domains with slightly different domain sizes. A description of the RCM settings of physical parameterizations and geographical coordinates is available from Iizumi *et al*. [23]. The initial and lateral boundary conditions of the RCMs for the present and future climate were obtained from the MIROC-H outputs in the twentieth century (20C3M) and A1B scenario experiment, respectively. Daily outputs of seven climatic variables for two 20 year periods (1981–2000 and 2081–2100) were available for all RCMs. Thereby, the climate change scenarios derived from the RCMs accounted for changes in climate variability and wet/dry spells as well as for mean climate change.

### (c) LARS-WG weather generator

We used a stochastic weather generator, the LARS-WG version 5 [51], available at http://www.rothamsted.bbsrc.ac.uk/mas-models/larswg.php. The LARS-WG is based on the series approach [52] and produces daily time series of 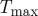, 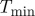, Pr and SR at a specific site based on a set of parameters for probability distributions of climatic variables and correlations between them derived from observed daily weather data at a given site for a long-term period. In the LARS-WG, probability distributions of climatic variables are modelled by using flexible semi-empirical distributions. The LARS-WG has proven adequacy in simulating daily values and extreme events across diverse climates [12,53]–55]. By modifying parameters for distributions at a given site using changes in climatic variables derived from a climate model, the LARS-WG can generate local-scale daily climate change scenarios that can be used as climate inputs for process-based impact models (e.g. [10]).

### (d) Estimation of relative humidity and wind speed

We separately generated daily mean values of RH and WS that are essential for the estimation of potential evapotranspiration (ET_0_) because the current version of the LARS-WG does not generate these climatic variables. Although the LARS-WG generates ET_0_ values using the Priestley–Taylor method [56], the methodological bias in ET_0_ is known. Indeed, the Priestley–Taylor method underestimates ET_0_ in winter and overestimates it in mountainous and coastal areas in summer under a humid climate, compared with the Penman–Monteith method [57]. Therefore, ET_0_ from the LARS-WG is not suitable for applications in Japan under a humid climate, specifically in summer (e.g. rice panicle temperature estimation; [58]).

Daily RH values were generated using the WXGEN weather generator [59], which uses a triangular distribution. Monthly mean, maximum and minimum RH values at a given site calculated from the observed data were the parameters for the distribution. For each site, the monthly mean RH value varied with time, whereas the maximum and minimum RH values for a month were fixed to be the maximum and minimum values in the month for the period 1981–2000 and did not change with time. Daily RH values from the WXGEN were conditioned on daily wet/dry conditions derived from the LARS-WG.

Daily WS values were independently generated from other climatic variables using a modified exponential distribution [60]. Namely, 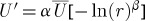, where *U*^′^ is the daily mean wind speed (m s^−1^), 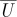 is the monthly mean wind speed (m s^−1^), *r* is a random number between 0 and 1, and *α* and *β* are parameters. We set (*α*,*β*)=(1.1,0.55) for the warm season (May–October) and (1.0, 0.55) for the cold season (November–April) for all sites based on the preliminary analysis results.

## 3. Performance of weather generators

### (a) Statistical tests

To evaluate the performance of WGs in simulating the observed statistical features of climatic variables, we used two types of statistical test, the two-sample Kolmogorov–Smirnov (K-S) test and Student's *t*-test. The K-S test was used to compare the whole probability distributions of climatic variables between two samples (i.e. the observed and generated data). The K-S test is a non-parametric test of one-dimensional distributions across two samples and has no restrictions on the shape of distributions. The null hypothesis of the K-S test is that two samples are drawn from the same distribution. The *t*-test was used to compare means of two samples with the assumption that two samples have the same variance. The *t*-test can be applied to the samples that are not normally distributed if the sample sizes are sufficiently large [61]. The null hypothesis of the *t*-test is that the means of two samples are equal. We computationally implemented these tests as described in Press *et al*. [61] and used them for analysis.

For each site, we compared the seasonal distributions of the wet and dry series (four tests for each type of series, wet or dry). The seasonal distributions correspond to the distributions for December–January–February (DJF), March–April–May (MAM), June–July–August (JJA) and September–October–November (SON). In this study, the wet day was defined as the day with daily precipitation greater than or equal to 1 mm d^−1^. In addition, we compared the monthly distributions and means of daily values of seven climatic variables (12 tests for each climatic variable and each type of test, K-S or *t*-test). The significance level was set to 1 per cent.

**Figure 2.**
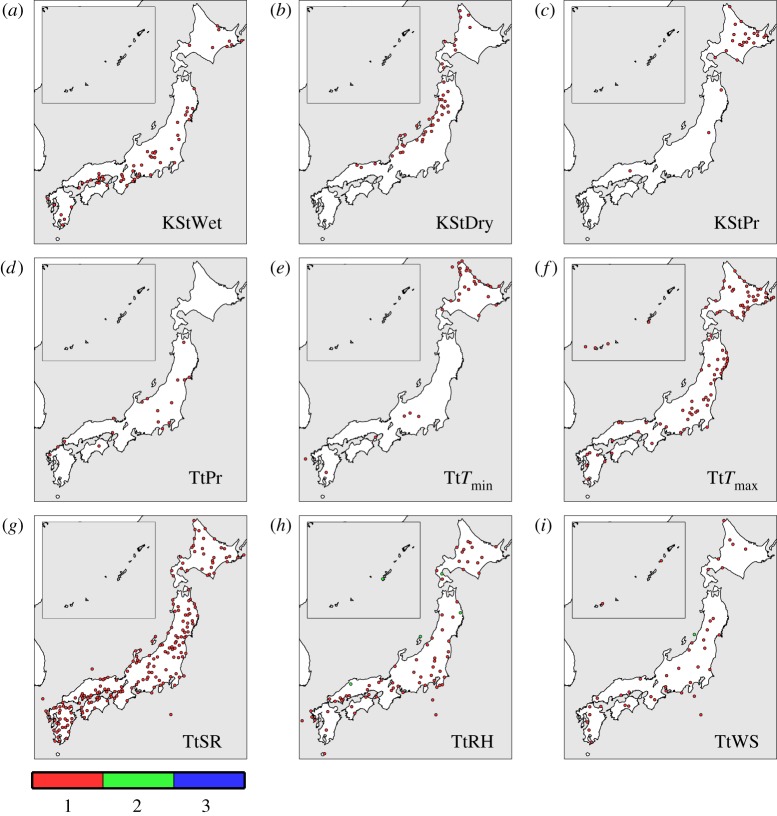
Sites with statistically significant differences between the generated and observed data in the K-S test for the seasonal distributions of (*a*) the wet series, (*b*) the dry series, (*c*) the K-S test for the monthly distribution of the daily precipitation, and the *t*-test for monthly means of daily (*d*) precipitation, (*e*) minimum temperatures, (*f*) maximum temperatures, (*g*) solar radiation, (*h*) relative humidity and (*i*) wind speed. The colour corresponds to the number of significant test results at a site.

The null hypothesis was not rejected in most sites (i.e. the generated data matched the observed ones; table 2). Thus, we here focus on the remaining exceptional sites. In statistical hypothesis testing, the test could incorrectly reject the null hypothesis with the probability equal to the significance level, even when the null hypothesis is in fact true (known as a false positive; [3]). In figure 2, the geographical distribution of sites with the significant differences is shown. There were no significant differences in the K-S tests for the monthly distributions for 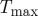, 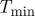, SR, RH and WS. We calculated the percentages of tests indicating a significant difference and summarized them in table 2. If we take the K-S test result for Pr as an example, the percentage of tests indicating exactly one significant difference is 2.5 per cent. Considering that 12 tests were performed for each site, the percentage of tests indicating exactly one significant difference per test is 0.2 per cent. This number is less than the expected false positive rate (1%) and acceptable from the point of view of the significance level we set. Similar results were found for the K-S test for RH and the *t*-test for 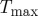, 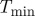, Pr, RH and WS (table 2). For these climatic variables, there were no biases in the spatial distribution of sites with a significant difference (figure 2), supporting the belief that the significant differences for these climatic variables are likely to be false positive results.

**Table 2.** Fraction of tests indicating a significant difference in the K-S test for the seasonal distributions of the wet and dry series and the monthly distributions of the daily precipitation and relative humidity, and in the *t*-test for the monthly means of the daily maximum and minimum temperatures, precipitation, solar radiation, relative humidity and wind speed (in per cent).

	K-S test				*t*-test					
no. of significant test results	wet	dry	Pr	RH	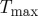	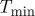	Pr	SR	RH	WS
0	93.5	94.9	97.5	99.8	89.8	96.4	98.1	77.1	91.9	95.5
1	6.5	5.1	2.5	0.2	10.2	3.6	1.9	22.9	7.6	4.4
2									0.5	0.1
sum	100.0	100.0	100.0	100.0	100.0	100.0	100.0	100.0	100.0	100.0

**Figure 3.**
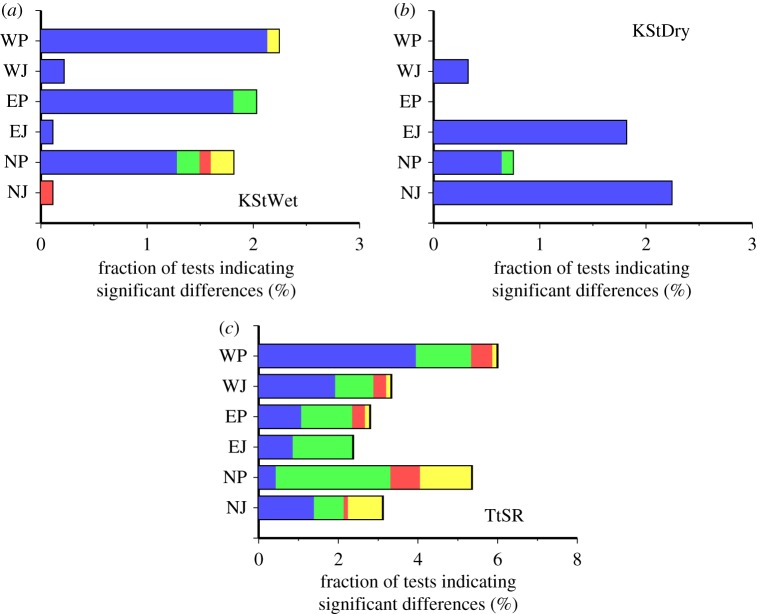
Fraction of tests indicating at least one significant difference in the K-S test for (*a*) the wet series, (*b*) the dry series or *t*-test result for (*c*) solar radiation for each season and each area in Japan (in per cent). The sum of these values corresponds to the values in table 2. There were no significant differences for the southwestern area (SW). Blue regions, DJF; green regions, MAM; red regions, JJA; yellow regions, SON.

For the K-S test for the wet and dry series and *t*-test for SR, the percentage of tests with exactly one significant difference per test is greater than the expected false positive rate (1.6%, 1.3% and 1.9% for the wet, dry series and SR, respectively). Figure 2 shows the sites with a significant difference distributed on the Pacific Ocean side areas (NP, EP and WP) for the wet series and on the Japan Sea side areas (NJ and EJ) for the dry series. The most significant differences in the K-S test for the wet and dry series occurred in winter (DJF; figure 3). As a major portion of the precipitation is produced by the winter monsoon (northwesterly wind from Eurasia) with a rich vapour supply from the Japan Sea, a clear contrast in the number of wet days between the two areas is formed in winter [23]. As a result, the observed wet series on the Pacific Ocean side areas and dry series on the Japan Sea side in winter are very short (less than 4 days at most sites). This makes the K-S test result sensitive to small differences in the number of wet or dry series between the observed and generated data.

Most sites with significant differences in the *t*-test for SR coincidentally showed significant differences in the K-S test for the wet or dry series (figure 2). If we removed the sites with significant differences in the K-S test for the wet and dry series, the percentage of tests with exactly one significant difference per test for SR is less than the expected false positive rate. However, many significant differences in the *t*-test for SR were observed in spring (MAM) as well as in winter (figure 3). This suggests that the differences in monthly mean SR between the observed and generated data are not only associated with the short wet (dry) series in winter but also other unknown factors.

### (b) Quantile–quantile plots at selected sites

**Figure 4.**
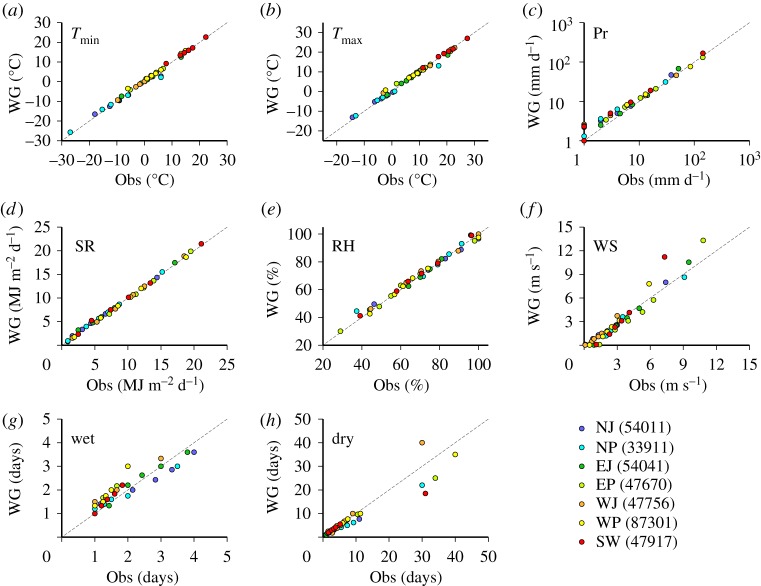
Q–Q plots of the observed and generated values of daily (*a*) minimum temperatures, (*b*) maximum temperatures, (*c*) precipitation, (*d*) solar radiation, (*e*) relative humidity, (*f*) wind speed, (*g*) wet series and (*h*) dry series for seven selected sites in winter (DJF). The quantiles between 0% and 100% for every 20% were calculated from 20 years of samples for the observed data and from 50 years of samples for the generated data.

**Figure 5.**
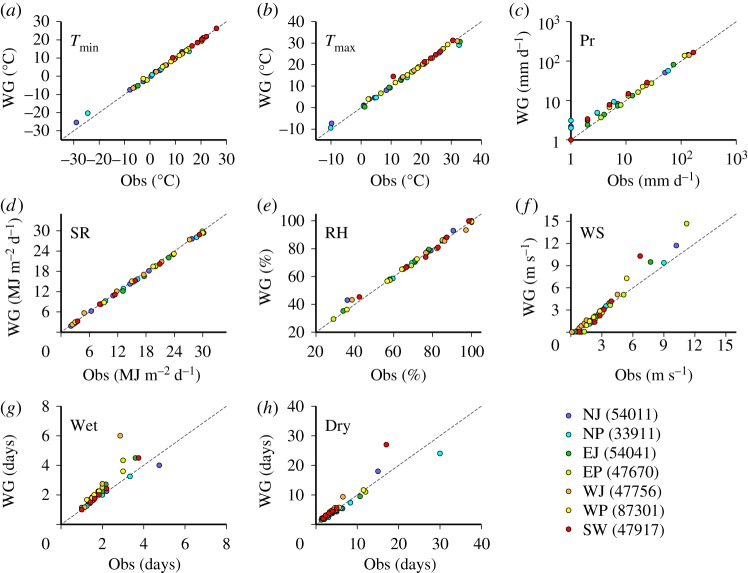
Same as figure 4 but for spring (MAM).

Following statistical tests, we visually checked the correspondence between the observed and generated data at selected sites, using quantile–quantile (Q–Q) plots for each season. A site that had many significant differences was selected for each area, as listed in table 3. For instance, site 33911 showed significant differences in the K-S test for the wet series and *t*-test for 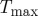 and SR. Considering the comparatively frequent occurrence of significant difference in winter (DJF) and spring (MAM), the Q–Q plots for these seasons are shown in figures 4 and 5. For most climatic variables, the correspondence in quantiles between the observed and generated data is good. A similar level of correspondence was observed for other seasons.

**Table 3.** Seven selected sites used for seasonal Q–Q plots.

code	name	area	longitude (^°^)	latitude (^°^)	altitude (m)	significant test result(s)
54011	Awashima	NJ	139.252	38.462	4	KStDry
33911	Ichinoseki	NP	141.125	38.933	32	KStWet, Tt 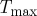 , TtSR
54041	Hagizaki	EJ	138.512	38.330	58	TtSR
47670	Yokohama	EP	139.652	35.438	39	TtPr, TtRH
47756	Tsutama	WJ	134.008	35.063	146	KStWet, KStPr, TtSR
87301	Kakuto	WP	130.810	32.047	228	KStWet, Tt 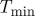 , TtRH
47917	Iriomotejima	SW	123.748	24.385	9	Tt 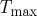

The discrepancies in quantiles for 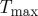, 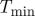, SR and RH in winter and spring are small even though significant differences in the *t*-test were found for either site. Small discrepancies in low-order quantiles were found for Pr but such discrepancies are likely to have little effect on impact model simulations. At site 47756, there are comparatively large and significant differences in high-order quantiles for the wet series in spring (figure 5*g*). However, the exact difference in the highest quantile between the observed and generated wet series in spring is just 3 days. While larger discrepancies in the highest quantile were found for the dry series in winter, such differences might not be a source of large error in impact model simulations, such as crop models, because the occurrence of such discrepancies is limited to the Japan Sea side areas (EJ and NJ) in winter (figure 3*b*).

## 4. ELPIS-JP dataset description

### (a) Scenario generation procedure

The dataset of local-scale daily climate change scenarios for Japan, ELPIS-JP, consists of two types of daily time-series data at 938 sites in Japan: (i) the transient scenarios for the period 1981–2091 using the CMIP3 multi-GCM outputs under three emission scenarios (A1B, A2 and B1) and (ii) the time-slice scenarios for two time points that represent the periods 1981–2000 and 2081–2100 using the S-5-3 multi-RCM outputs based on the MIROC-H (20C3M and A1B). Seven climatic variables (*T*_ave_, 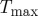, 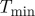, Pr, SR, RH and WS) are available. *T*_ave_ was calculated by averaging 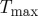 and 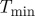. For each site, 50 sets of daily weather data from the WGs (referred to as ‘ensembles’) were available for each climate model, each emission scenario and each type of scenario (transient or time slice). These ensembles are possible representations of daily weather and equivalent to each other as they were drawn from the same distributions of climatic variables. In total, 1100 scenarios, consisting of 50 ensembles×(3 emissions×6 GCMs+2 emissions×2 GCMs) are available for each site for the transient scenarios. The number of the time-slice scenarios is 300 (consisting of 50 ensembles×3 RCMs×2 emissions).

Although a 30 year period has generally been used in previous studies to estimate WG parameters, we adopted the comparatively shorter period (20 year period 1981–2000) to cover the area of Japan as densely as possible, considering that most AMeDAS sites began their observation during or after 1979. Another reason is that the RCM outputs of the present climate simulation are available only for the 20 year period.

The LARS-WG cannot provide data with long-term variations (e.g. trend) because of the stationary assumption [62]. Lazzarotto *et al*. [14] assumed the linear trend in the changes of climate from the baseline period to 2100 and generated the transient scenarios throughout the twenty-first century with the trend using the LARS-WG. This linear trend assumption may be an oversimplification because state-of-the-art GCMs with appropriate initial conditions have the ability to predict the trend in climate at a decadal scale [63]. To account for the nonlinear trend simulated by the GCMs in the transient scenarios, we set the changes in climatic variables calculated from a 20 year period centred on an intended year, relative to the period 1981–2000, for the WGs year by year to annually modify parameters for distributions. In other words, the changes in climatic variables calculated from the period 1971–1990 were used to generate data for 1981. With this setting, it is feasible to generate data with time variations holding the stationary assumption. This is an approach for climate change scenario generation using WGs that rely more on information derived from GCMs than the other approaches [14]. To that end, the exact final year of the transient scenarios is 2091, which represents the period 2081–2100. No trend was considered for the time-slice scenarios owing to the limited RCM simulation period.

### (b) Features and limitations

Both types of scenarios, transient or time slice, are important but used for different purposes. The transient scenarios are preferable for quantifying the uncertainty of future impacts associated with GCMs and emission scenarios. In addition, transient climate scenarios are required for impact model simulations that need long-term time integration, such as vegetation succession, soil organic matter decomposition and soil erosion.

For the time-slice scenarios after dynamical downscaling with RCMs, more detailed geographical patterns of projected change are available. Spatially detailed climate information is central for impact model simulations that have high sensitivity to topography, e.g. orographic precipitation, snow accumulation in complex terrain and heat wave associated with airflow over mountains (e.g. foehn; [45]). The time-slice scenarios are based on RCMs and generally achieve better representation of extreme events (e.g. high-order quantile of daily precipitation; [23]) than the GCMs. These scenarios are more suitable for assessment of impacts for systems sensitive to extreme events (e.g. crop yield responses to high-temperature stress at anthesis; [51]) and application for disaster studies.

**Figure 6.**
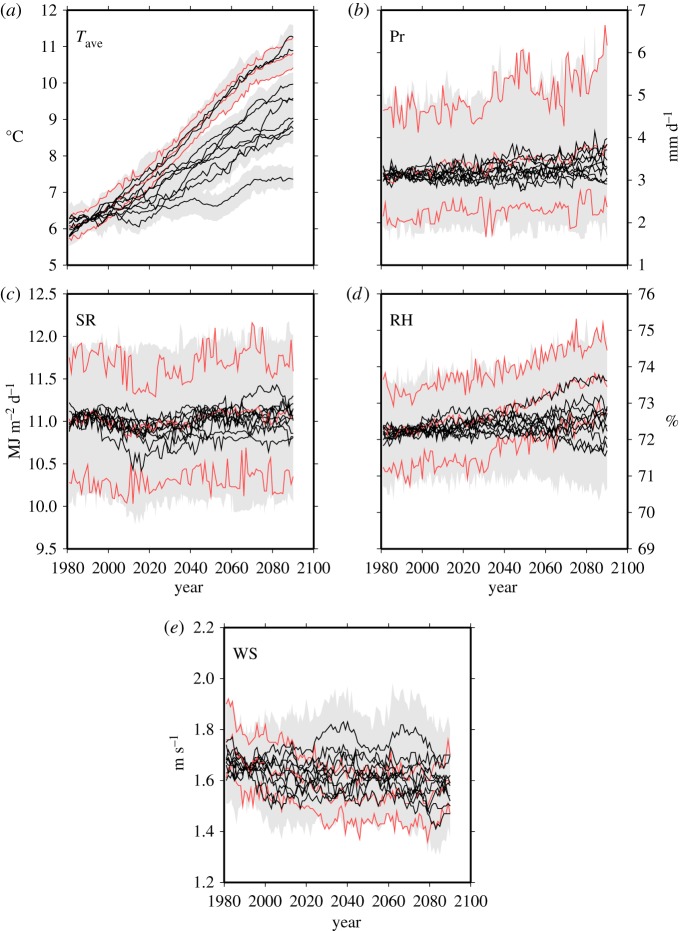
Example of the transient scenarios at site 47420 (Nemuro: 43.33^°^N, 145.585^°^E, 25 m) for annual means of (*a*) the daily mean temperature, (*b*) precipitation, (*c*) solar radiation, (*d*) relative humidity and (*e*) wind speed under A1B. The black line and the grey shaded area indicate median and 99% interval of 50 ensembles from the WGs for each GCM, respectively. The red line indicates the corresponding values for the MIROC-H (only the mean climate change).

The advantages of one type of scenario are disadvantages of another type of scenario. For the transient scenarios, the geographical pattern of projected change is spatially coarser than that of the time-slice ones even for the MIROC-H, which has the finest grid interval among the GCMs (figure 6). Changes in climatic variability and wet/dry spells are not accounted for except for the scenarios using the MIROC-H (A1B). Changes in intensity and frequency of extreme events might be smaller than those for the time-slice ones. The transient scenarios that accounted for only the mean climate change of the MIROC-H are available in the dataset to enable users to compare the transient scenarios with and without the incorporation of changes in climate variability and wet/dry spells. For the time-slice scenarios, the relative disadvantages compared with the transient ones are their inapplicability to assessments that need to simulate long-term time evolution of quantity of interest, fewer numbers of GCMs and emission scenarios, and no consideration of time variations.

### (c) Note for users

The ELPIS-JP dataset is open for scientific communities and efforts to make the dataset downloadable from the Internet have been planned. We describe known issues and inappropriate applications of climate scenarios to facilitate uptake and better understanding by the impact community, taking the ELPIS-JP dataset as an example (see also the UK climate projections: http://ukclimateprojections.defra.gov.uk/content/view/1793/521/).

**Figure 7.**
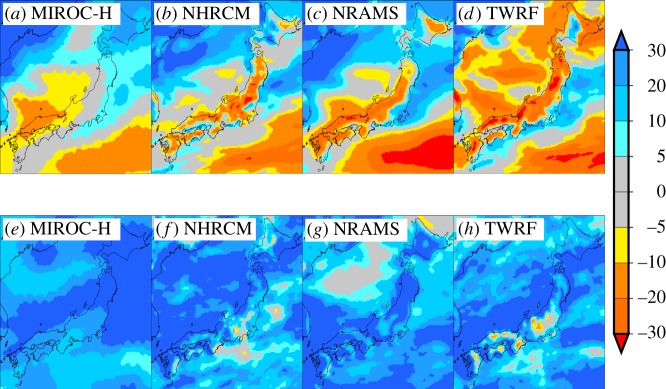
Percentage changes in seasonal mean precipitation for (*a*–*d*) winter (DJF) and (*e*–*h*) summer (JJA) over Japan for the period 2081–2100, relative to 1981–2000, from the MIROC-H and three RCMs (NHRCM, NRAMS and TWRF).

— The daily site time series are not spatially correlated. For regional assessment, daily site data cannot be spatially averaged to produce aggregated data for a user-defined area. Users should simulate the impact site by site from a selected region, and then spatially aggregate impact results.
— The ensembles of WG climate change scenarios for a given GCM (or RCM) represent the uncertainty associated with interannual climate variability. They do not represent the uncertainty of climate projections associated with climate models or emission scenarios.
— Transient climate change scenarios do not coincide with observed historical weather, and should be considered as plausible samples of 100 years of weather data. That is why users cannot compare the simulated quantity of interest in a specific year (e.g. crop yield in 1981) with observed data in that particular year. However, the use of long-term statistics is appropriate for comparison.
— Users should not limit their analysis to a single GCM or RCM. There is no clear rationale to select one climate model for impact assessment. The differences in projected changes among the RCMs are not negligible even when they are using the same GCM as the initial and boundary conditions (figure 7). The whole ensemble should be used to assess the uncertainty of future impacts (see IPCC Expert Meeting Report [64] for discussion on performance metrics for GCM selection and weighting). Users should be aware that CMIP3 and S-5-3 ensembles did not systematically explore all uncertainty of climate projections.
— Owing to nonlinearity responses to environmental and climatic variations in process-based impact models, users should avoid interpolating between two values of simulated impacts using two specific climate scenarios representing, for example, 10 and 90 per cent quantiles to estimate the impact at an arbitrary value of projected changes. This might produce a large discrepancy between simulated and interpolated impacts (see [6] for crop model case).

## 5. Conclusions

We provided a dataset of local-scale daily climate change scenarios for Japan, ELPIS-JP, following publication of ELPIS for Europe. For each of 938 sites in Japan, ELPIS-JP provides an ensemble of 50 daily time-series data based on the climate projections from the CMIP3 and S-5-3 multi-model ensembles. The use of ELPIS-JP allows assessment of the impact resulting not only from mean changes in climate but also from changes in climatic variability, including changes in wet/dry spells and extreme events. Uncertainty in the prediction of future impacts associated with the uncertainty in climate models and emission scenarios can be also estimated. This dataset is designed to be used in conjunction with process-based impact models and offers a flexible framework for probabilistic assessments of future impacts and adaptation at a local scale in Japan. This study highlights the efforts to interpret information from climate models to assess the impact through the development of climate change scenarios using WGs and summarizes the features and limitations of the scenarios: these efforts go beyond mere validation of existing WGs in a specific area.

The performance of WGs, the LARS-WG and, in part, the WXGEN, was assessed using the statistical tests and Q–Q plots. The overall skills of the WGs are good. The Q–Q plots for the selected sites showed that the discrepancies between the observed and generated data are small in most cases, even when significant differences were found. Most discrepancies are likely to have little effect on the outcome of impact analysis.

The spatial interpolation of WG parameters is an area not explored here, which will allow impact assessment at any selected location in Japan (similar to the UK; [10]). However, in Japan, high-resolution gridded daily datasets are available for Pr [65], *T*_ave_, 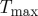, 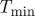 and SR [66], as well as the gridded monthly climatology [67]. The use of such datasets might be a more preferable option than spatial interpolation of WG site parameters. One area for further improvement of WGs would be incorporation of spatial correlation between sites [68,69].
